# A set-based association test identifies sex-specific gene sets associated with type 2 diabetes

**DOI:** 10.3389/fgene.2014.00395

**Published:** 2014-11-12

**Authors:** Tao He, Ping-Shou Zhong, Yuehua Cui

**Affiliations:** ^1^Department of Statistics and Probability, Michigan State UniversityEast Lansing, MI, USA; ^2^Division of Medical Statistics, School of Public Health, Shanxi Medical UniversityTaiyuan, China

**Keywords:** *p*-value combination, gene-based analysis, pathway-based analysis, sex-specific analysis, type 2 diabetes

## Abstract

Single variant analysis in genome-wide association studies (GWAS) has been proven to be successful in identifying thousands of genetic variants associated with hundreds of complex diseases. However, these identified variants only explain a small fraction of inheritable variability in many diseases, suggesting that other resources, such as multilevel genetic variations, may contribute to disease susceptibility. In this work, we proposed to combine genetic variants that belong to a gene set, such as at gene- and pathway-level to form an integrated signal aimed to identify major players that function in a coordinated manner conferring disease risk. The integrated analysis provides novel insight into disease etiology while individual signals could be easily missed by single variant analysis. We applied our approach to a genome-wide association study of type 2 diabetes (T2D) with male and female data analyzed separately. Novel sex-specific genes and pathways were identified to increase the risk of T2D. We also demonstrated the performance of signal integration through simulation studies.

## 1. Introduction

Advancements in microarray and next generation sequencing technologies enable people to find more and more genetic variants, including small variations in single nucleotide polymorphisms (SNPs) and large variations, such as indel and copy number variation. Genome-wide association studies (GWAS), which focus on the association between SNPs and complex diseases, have been proven to be a powerful tool to unveil the genetic secret of complex diseases. However, the identified SNPs can only account for a small fraction of heritability in many diseases, motivating people to develop more advanced statistical methods and novel models with the hope to find the missing heritability. Among the large efforts being pursued, it is natural to consider gene-set or pathway-based analysis given that genes in a pathway or network tend to work coordinately to fulfill their tasks. The subtle effects in multiple SNPs can be combined so that the joint signal in a gene set could be potentially boosted. A variety of public resources are available to create the gene-set, such as Kyoto Encyclopedia of Genes and Genomes (KEGG) (Kanehisa, 2000), Reactome (Joshi-Tope et al., 2005) and Gene Ontology (Ashburner et al., 2000). Signal-based combination methods have been broadly applied in this context and can be classified into two categories: the one-step approach and the two-step approach. The former one treats all SNPs equally and combines effects of all SNPs within a certain pathway to come up with a total signal. The latter one firstly obtains a gene-score by combining SNP effects in a gene, then constructs a higher hierarchical test statistic to score a gene-set (or pathway) using the previously achieved gene scores. In either case, the significance of a bigger set (e.g., a pathway) is evaluated using either asymptotic approach, or more practically, permutation techniques. However, the one-step approach might lose power since the association signals are equally considered in different genes without considering the hierarchical structure of a gene set.

Toward the signal integration, *p*-value combination is a popular one since *p*-value possesses several nice characteristics such as model-free, clear interpretation and standard scale. Different *p*-value combination methods were widely discussed in the literature, such as Fisher's method that summarizes signals using a transformation of the *p*-value product as the test statistic (Fisher, 1932), the truncated product (TP) method which considers the product of *p*-values less than a pre-defined threshold (Zaykin et al., 2002), the rank truncated product (RTP) method which employs top *R* most significant *p*-values with *R* being the pre-selected rank truncation threshold (Zaykin et al., 2006), and the adaptive rank truncation product (ARTP) method where the rank truncation point is adaptively selected from *T* truncation candidates *R*_1_, ···, *R_T_* to optimize the permutation *p*-value of the test statistic (Hoh et al., 2001; Dudbridge and Koeleman, 2004). Furthermore, *p*-value combination can be applied either in one-step combination or two-step combination. Although the one-step ARTP method (e.g., Hoh et al., 2001; Dudbridge and Koeleman, 2004) is more flexible to the choices of the truncation point compared to other one-step truncation methods, it uses one single threshold for all the genes in a pathway, disregarding of their individual sizes and linkage disequilibrium (LD) structures, hence may not be reasonable in practice. In fact, an overestimated truncation might dilute the signal, while underestimation will lead to major signal loss. To overcome the limitation of the one-step analysis, a two-step ARTP model was developed Yu et al. (2009). However, there is no unified criterion for the choice of *T*. In addition, as a permutation-based method, ARTP is computationally expensive to achieve a small *p*-value, and this burden could be much heavier when *T* is chosen to be large.

These limitations motivate us to consider a two-step analysis while maintaining the hierarchical genetic structure in a gene set. Li et al. (2011) proposed a one-step *p*-value combination approach to infer pathway regulations in an eQTL mapping framework. By introducing the hierarchical pathway structure, we extend this method to a two-step one under the GWAS framework and name it as scaled chi-square combination (SCC) method. We propose to combine individual SNP *p*-values in a lower genetic system (e.g., a gene) in the first step, then summarize the gene signals in a higher system (e.g., a pathway) in the second step, while considering the correlations among genetic variants in the SNP- and gene-level. This joint analysis could spark additional insights into pathway function on a disease trait that otherwise could be missed by looking at individual signals alone. Another advantage using SCC method is that there is no need for truncation, which also implies no information dropping. Besides, since *p*-value is derived based on a chi-square approximation, small *p*-values can be obtained which cannot be easily achieved by permutation-based methods.

Examples of other two-step methods also include gene set scan (GSS) (Schaid et al., 2012) and modified gene set enrichment analysis (MGSEA) (Wang et al., 2007). In GSS, each gene is firstly scored using average SNP signals, which are individually evaluated via a score statistics from a regression model. Then a pathway signal is scored by the weighted “average” signals from genes, and a normal distribution is assumed to compute the *p*-value of a pathway. While in MGSEA, the smallest *p*-value among all SNPs in a gene is chosen to represent the gene signal. Pathways are then scored using weighted Kolmogorov-Smirnov like running sum statistic, and the significance of a pathway is evaluated based on permutation approach. Obviously the GSS method is limited by taking average when large number of noisy SNPs are present.

The article begins with a detailed description of using Fisher's combination statistic to score genes and pathways, and the Satterthwaite approximation of the null distribution of the combined *p*-values. Section 3 provides real data analysis on two type 2 diabetes (T2D) cohorts data sets. In section 4 we demonstrate the utility of SCC via extensive simulations and compare it to other two-step methods (e.g., ARTP and MGESA) under a variety of scenarios, followed by the discussion in section 5.

## 2. Statistical methods

### 2.1. Combining and approximating

Assume *L* tests are conducted for the association between a disease trait and *L* SNPs in a given genetic system, and let *p*_1_, *p*_2_, ···, *p_L_* be the *p*-values for the *L* individual tests. For example, if we want to test whether a gene (consisting *L* SNPs) is associated with a disease trait, the gene would be the system and individual SNP is the unit; pathway and gene would be the system and unit respectively when testing the association between a disease trait and a pathway containing *L* genes. Define *W_i_* = −2log *p_i_*. Under the null hypothesis of no genetic effect, each of the *L p*-values is uniformly distributed and *W_i_* ~ χ^2^_2_ for *i* = 1, ···, *L*. If we assume the *L* tests are independent, the Fisher's combined statistic $Z=\sum_{i\;=\;1}^{L}W_{i}\sim \chi_{2L}^{2}$ under the global null hypothesis of no genetic effect in the genetic system.

Since units in a genetic system tend to work coordinately, they are more or less correlated. Hence the *L p*-values are not independent and the chi-square distribution with 2*L* degrees of freedom (*df*) in Fisher's method may not be true. To adaptively account for the combined effect of *L* correlated χ^2^_2_ distributions, Satterthwaite's approximation can be introduced, where a scaled chi-square distribution is used to approximate the null distribution of *Z*, i.e.,

(1) $$Z=\sum_{i=1}^{L}W_{i}\dot{\sim }a\chi_{d}^{2},$$

and the scale parameter *a* and the *df* parameter *d* are chosen by equating the first and second moments of the scaled chi-square distribution with the ones of *Z* under the null hypothesis, respectively. Under the independence case, the means and variances of the statistic *Z* under the null are

$$\begin{array}{l} \text{E}(Z)=\text{E}\left(\sum_{i=1}^{L}W_{i}\right)=2L,\\ \text{Var}(Z)=4L+8\sum_{i<j}\rho_{ij}, \end{array}$$

where ρ_*ij*_ = Cov(*W_i_*, *W_j_*) is the correlation coefficient between the log-transformed *p*-values *W_i_* and *W_j_*. Solving the moment equations E(*Z*)≐E(*a*χ^2^_*d*_) = *ad* and Var(*Z*) ≐ Var(*a*χ^2^_*d*_) = 2*a*^2^*d*, we have

(2) $$\hat{a}=1+\frac{2\sum_{i<j}\rho_{ij}}{L},\;\hat{d}=\frac{2L^{2}}{L+2\sum_{i<j}\rho_{ij}},$$

When the *L* units in a genetic system are completely independent, i.e., ρ_*ij*_ = 0 for all *i* ≠ *j*, the Satterthwaite's approximation (*â* = 1, $\hat{d}$ = 2*L*) is exactly the same as the distribution of the Fisher's combined statistic which assumes independence. When the *L* units are perfectly dependent in the meaning of ρ_*ij*_ = 1 for all *i*≠ *j*, the approximation degenerates to *â* = *L*, $\hat{d}$ = 2. Beyond these extreme cases, the scaled chi-square approximation has much flexibility to account for the correlation structure among the units.

### 2.2. Gene set scoring

In the following we describe how to score genetic unit in each level. Assume the pathway of interest consists of *K* genes, where the *k*th gene consisting *n_k_* SNPs (1 ≤ *k* ≤ *K*) and (*p^k^*_1_, ···, *p^k^_n_k__*) are *p*-values of those SNPs. Let Γ_*k*_ be the correlation matrix of the transformed *p*-value vector **w**^*k*^ = (*w^k^*_1_, ···, *w^k^_n_k__*), which represents the correlation structure among the *n_k_* SNPs within the *k*th gene. For each gene, a combined score $z_{k}^{gene}=\sum_{m=1}^{n_{k}}w_{m}^{k}$ could be obtained. Together with the shape parameter (*â*_*k*_, $\hat{d}$_*k*_) estimated in (2), a gene-based *p*-value

$$p_{k}^{gene}=P\left(z_{k}^{gene}>{\hat{s}}_{k}\chi_{{\hat{d}}_{k}}^{2}\right),\;1\leq k\leq K$$

can be obtained. This finishes the signal combination of the first step.

Let **w**^*gene*^ = (*w^gene^*_1_, ···, *w^gene^_K_*) be the -2log transformed *p*-value vector for the *K* genes obtained in the first step, and Γ is the corresponding correlation matrix. In the second step, the pathway can be scored as $z^{path}=\sum_{m=1}^{K}w_{m}^{gene}$ and the shape parameters (*â*_2_,$\hat{d}$_2_) can be estimated in a similar way. Then a *p*-value of the pathway, which reflects its association strength with a disease trait, can be obtained by $p^{path}=P\left(z^{path}>{\hat{a}}_{2}\chi_{{\hat{d}}_{2}}^{2}\right)$. The problem remaining is to estimate the correlation matrices among *L* SNPs as well as among *K* genes, which is discussed in the next section.

### 2.3. Estimating correlation matrix

A permutation (or resampling) procedure is applied here to estimate the correlation matrix of a transformed *p*-value vector on a gene- or pathway-level. We first obtain the *L* dimensional transformed *p*-value vector **w**^(0)^ = {*w*^(0)^_1_, ···, *w*^(0)^_*L*_} through single-SNP testing based on the original data. Then we generate *B* datasets under the null hypothesis of no association between the SNPs and trait, by simply permuting the trait label while keeping the genotype fixed such that the correlation structure among SNPs/genes is not disrupted. Through each of the permuting process, we could obtain a new transformed *p*-value vector **w**^(*b*)^ (*b* = 1, ···, *B*). Then the correlation matrices Γ*_k_*, *k* = 1 ···, *K* and Γ can be estimated using the sample correlation matrices from the permuted data. Note that only one single layer of permutations is needed, and moderate permutations are needed to estimate the correlations in Γ_*k*_, *k* = 1 ···, *K* and Γ. Typically *B* = 200 is enough to obtain reasonable estimation of the correlation matices, compared to large number of permutations to obtain small *p*-values as implemented in the ARTP procedure.

## 3. Results

### 3.1. Real data analysis

A growing number of experimental evidence shows that there is gender effect related to type 2 diabetes (T2D). For instance, individual SNP test results indicate moderately differential signals between male and female population (Wu and Cui, 2013), and there also exists sex difference in the impact of T2D on coronary heart disease risk (Juutilainen et al., 2004). Therefore, we are specifically interested in identifying sex-specific pathways associated with T2D with the hope to gain novel insight into the disease etiology of T2D in different sex groups. We applied four different two-step methods including SCC, ARTP, MGSEA and GSS to two nested case-control cohort T2D datasets, the Nurses' Healthy Study (NHS) and the Health Professional Fellow-up Study (HPFS), which are part of the Gene, Environment Association Studies (GENVEA) (Cornelis et al., 2010). Please refer to Hu et al. (2001) and van Dam (2002) for more detailed information about the datasets. The raw datasets originally include 3391 female and 2599 male participants with European ancestry, respectively. Individuals with large proportion of missing SNPs (>10%) or large kinship relationship with others were removed. We also removed SNPs with minor allele frequency (MAF) <0.05 or deviation from Hardy-Weinberg equilibrium (*p* < 0.001) in controls. The final data set contains 672,105 SNPs in 3371 females (1572 cases and 1799 controls) and 671,669 SNPs in 2494 males (1161 cases and 1333 controls). SNPs that are within 50 kb up- and down-stream of a gene were assigned to the corresponding gene based on Human Genome Build 37.3. Totally 186 KEGG pathways was retrieved from the Molecular Signatures Database (MSigDB) covering 5015 genes using the Gene Set Enrichment Analysis package (Subramanian et al., 2005). The final data set contains 143,137 SNPs in female and 143,272 SNPs in male.

We conducted separated analysis in the two gender groups, in each of which SNP signals were combined together in two steps to represent the overall contribution of the pathway. Seven covariates (*p*-value < 0.05) was included into the model, including family history of diabetes among first degree relatives, reported high blood pressure at/before blood draw, reported high blood cholesterol at/before blood draw, total physical activity, body mass index, heme iron intake and glycemic load, to adjust for the covariates' effect when fitting a logistic regression model to test individual SNP effect. An additive gene action model was assumed when coding the SNP effect. The obtained *p*-values were then applied to the four methods, SCC, ARTP, MGSEA, and GSS following the recommended settings of each method.

**Figure 1:**
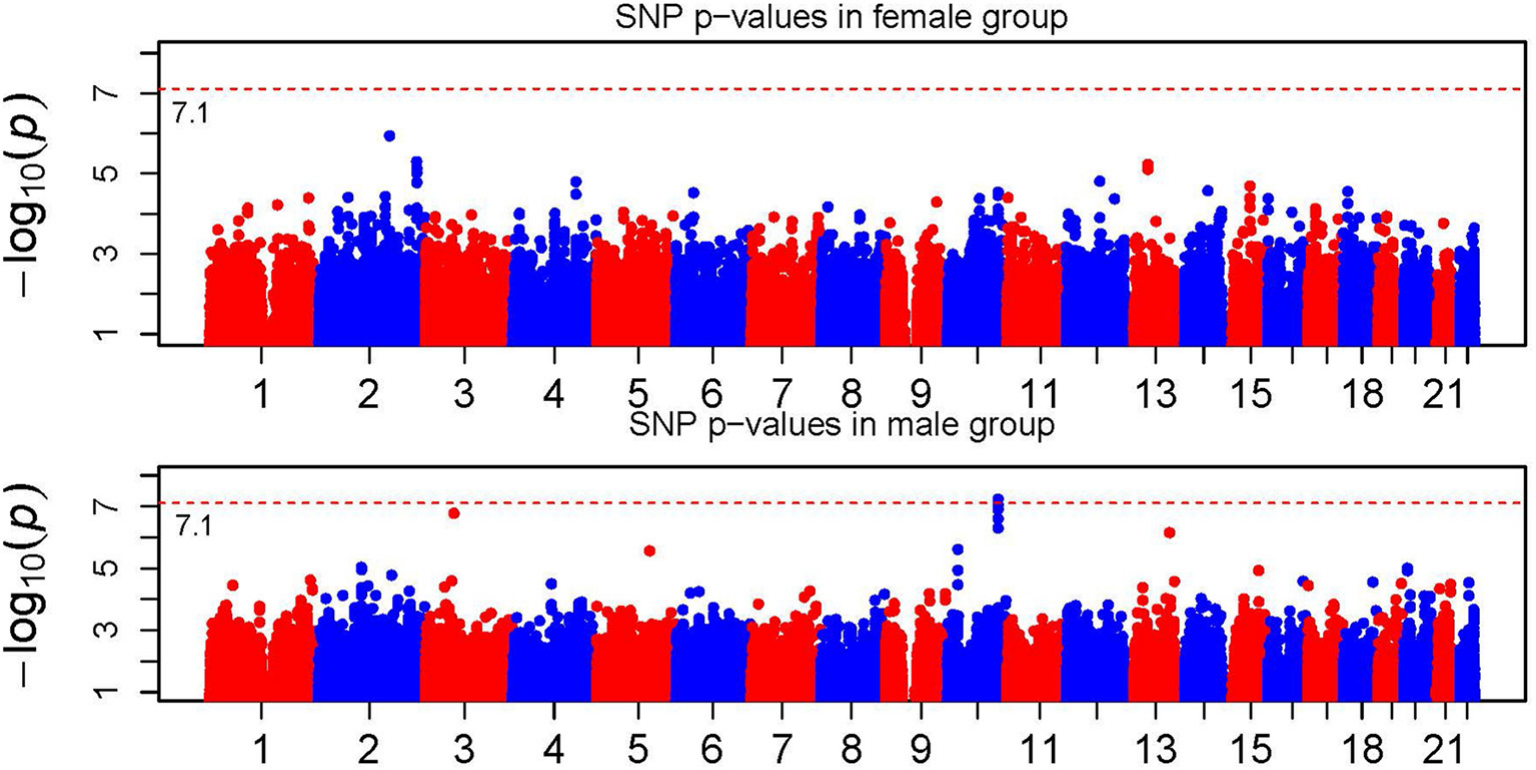
**Manhattan plot of single SNP *p*-values**. The horizontal dotted line represents the genome-wide significance threshold which is labeled in the figure.

Figure 1 shows the Manhattan plot of the single SNP signals across the 22 autosomal chromosomes for the female and male groups with the vertical axes represent the −log10 *p*-values. The dashed line corresponds to the genome-wide Bonferroni threshold. The overall pattern is quite similar in the male and female population with a few clear differences, especially on chromosome 2, 3, 4, 5, 10, and 13. The signals passing the genome-wide Bonferroni threshold located on chromosome 10 in the male group are from gene TCF7L2, one of the genes associated with T2D and being replicated in several GWAS studies (Grant et al., 2006; Sladek et al., 2007).

**Figure 2:**
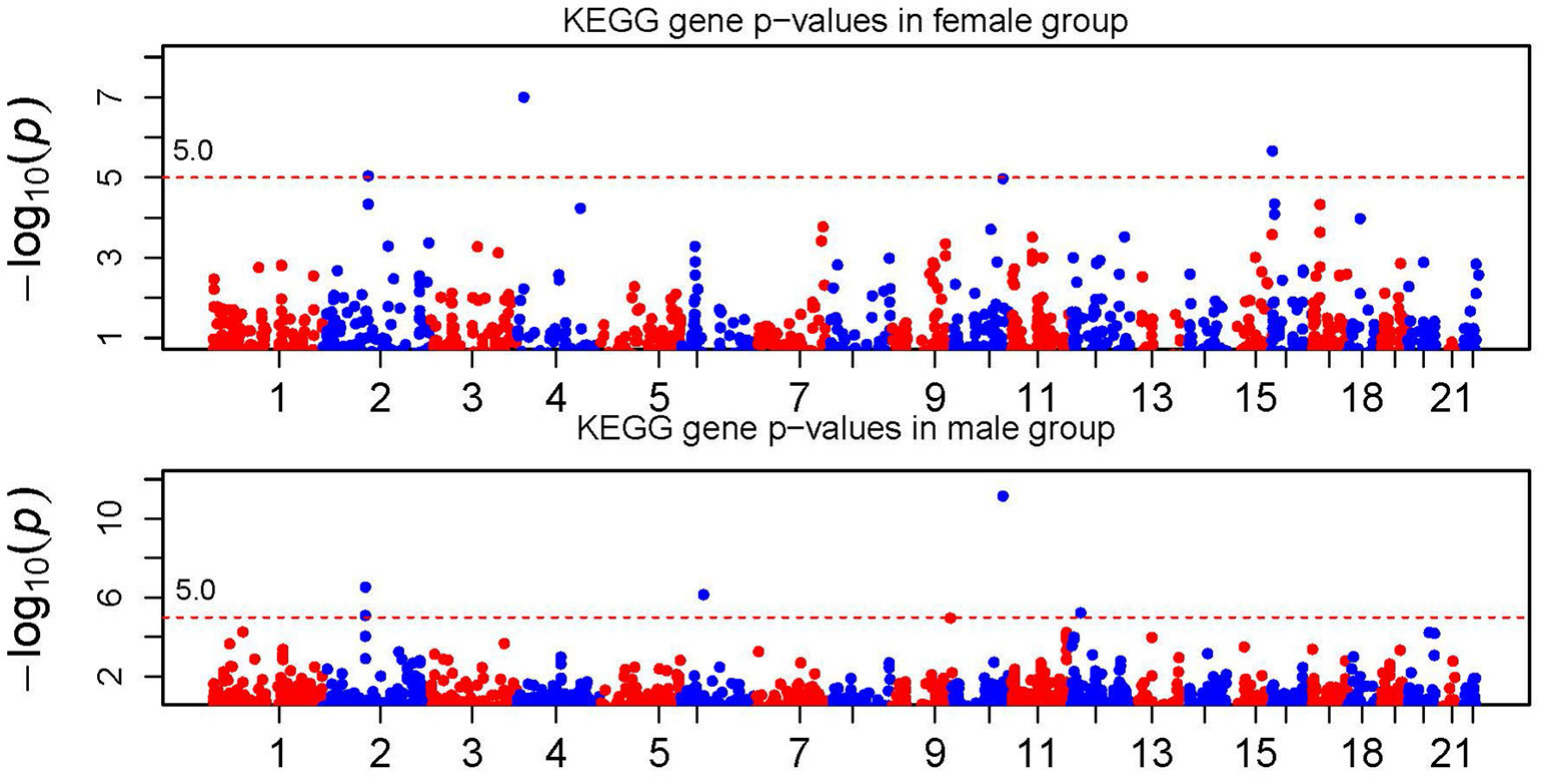
**Manhattan plot of KEGG gene *p*-values**. The horizontal dotted line represents the genome-wide gene-level significance threshold which is labeled in the figure.

Figure 2 shows the KEGG gene signals when combining SNP signals in a gene. Again the dashed line corresponds to the genome-wide Bonferroni threshold. Genes passing the Bonferroni threshold are IL18RAP (on chr2), BST1 (on chr4), TCF7L2 (on chr10) and PIGQ (on chr16) in the female population, and SNRNP200 and DUSP2 (on chr2), MCM3 (on chr6), ABO (on chr9), TCF7L2 (on chr10) and GYS2 (on chr12) in the male population. Again we observed gender difference in association signals at the gene level. It is worthy to note that although all SNPs in gene TCF7L2 in the female population do not show significance, the combined gene signal reached the genome-wide gene-level significance. This shows the power of signal combination to boost the association signal.

In a short summary, we observed significant gender difference in association signals at both the SNP and gene level. In addition, we found that the gene-level signal was more significant through signal combination. For example, the gene-level signal on chromosome 2 passed the genome-wide Bonferroni threshold (~ 10^−5^), while the SNP-level *p*-value failed. Under the scenarios where multiple causal SNPs with moderate or small effects are not significant individually, the gene-level signal can be potentially boosted through combination. This is one of the major advantages of using combined method. The top gene-level signals using the ARTP method with the recommended parameter settings, were partially similar to the results obtained using the SCC method (see Supplementary Table 1 for details). However, more permutations are needed to achieve small *p*-values by using the ARTP method which imposes more computation burden. Note that not all SNPs with strong signals in the SNP-level analysis can be mapped to a gene. Thus, it is not surprise why some locations have strong SNP signals but weak gene signals.

**Figure 3:**
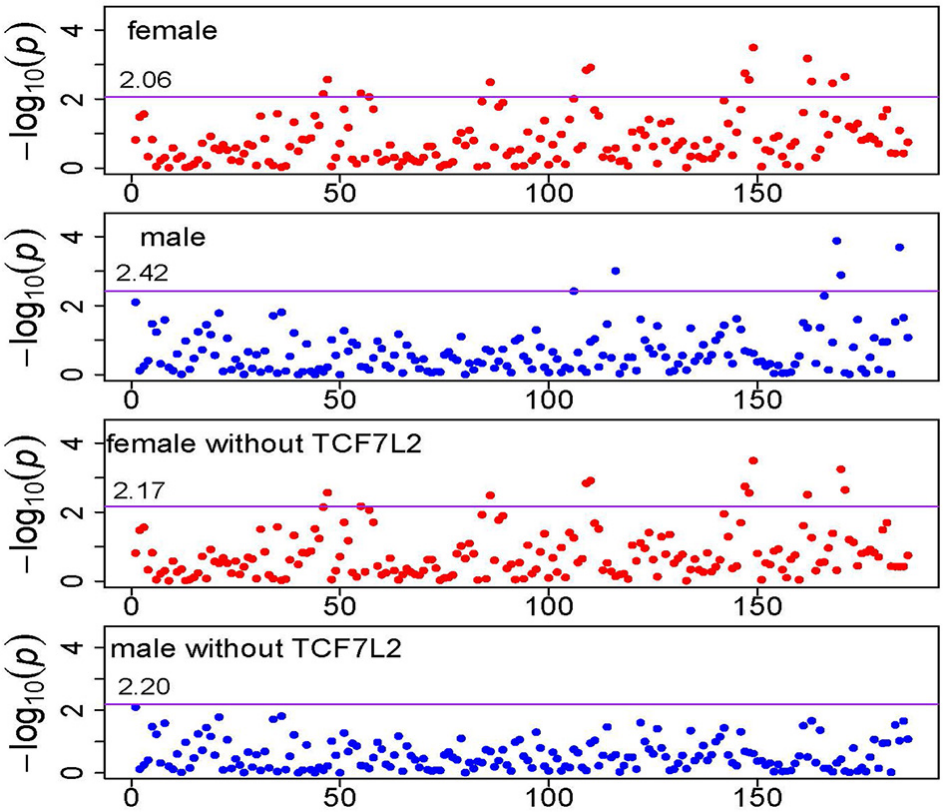
**Manhattan plot of KEGG pathway *p*-values**. The horizontal solid line represents the genome-wide pathway level significance threshold which is labeled in the figure.

Table 1 summarizes the significant KEGG pathways in female and male groups after the FDR control with *q*-value < 0.05 (Storey and Tibshirani, 2003). Total 13 pathways were enriched in female group and 5 were enriched in male group, with 3 in common. There is clear heterogeneity in pathway association between the two sex groups. Since TCF7L2 belongs to several enriched pathways (3 in female group, 5 in male group) and is widely recognized as a gene conferring risk of T2D, we conducted the same analysis but deleting this gene in all pathways. Figure 3 shows the pathway-level signals across 186 KEGG pathways with and without TCF7L2, in female and male groups, respectively, where the solid line represents the FDR threshold. It can be seen from Figure 3 that there is no significant change between pathway signals with and without gene TCF7L2 in female group, while the strong signals in male group are almost vanished after deleting the gene. This observation suggests potential difference in T2D etiology in the pathway level in each gender group. The significance of the pathways in the male group is largely dominated by gene TCF7L2.

**Table 1:** **List of enriched KEGG Pathways in Female and Male population**.

**KEGG pathway name**	**No. genes**	**Female**	**Male**
KEGG maturity onset diabetes of the young	25	✓	–
KEGG pathways in cancer	315	✓	–
KEGG TGF beta signaling pathway	86	✓	–
KEGG hedgehog signaling pathway	55	✓	–
KEGG type II diabetes mellitus	46	✓	–
KEGG melanoma	67	✓	–
KEGG sphingolipid metabolism	37	✓	–
KEGG type I diabetes mellitus	41	✓	–
KEGG MAPK signaling pathway	256	✓	–
KEGG one carbon pool by folate	17	✓	–
KEGG alpha linolenic acid metabolism	19	✓	–
KEGG thyroid cancer^*^	29	–	✓
KEGG arrhythmogenic right ventricular cardiomyopathy ARVC^*^	72	–	✓
KEGG adherens junction^*^	73	–	✓
KEGG basal cell carcinoma^*^	55	✓	✓
KEGG colorectal cancer^*+^	61	✓	✓

+ *Pathway signal decreased greatly when TCF7L2 gene was deleted in female*.

* *Pathway signal decreased greatly when TCF7L2 gene was deleted in male*.

The other three methods, ARTP, MGSEA, and GSS, all failed to detect any significant pathways after the FDR control (see Supplementary Tables 2–4). GSS gives *p*-values all greater than 0.08 for the 186 pathways. This is not surprising since GSS tends to lose power due to averaging when large number of noise variants are included (Schaid et al., 2012). The method could be benefitted by a SNP pre-selection (Wu and Cui, 2014). It should be noted that KEGG *type II diabetes mellitus* pathway, which is a manually annotated gene set, is only identified using the SCC method. This indicates the robustness of the SCC method.

## 4. Simulation study

To evaluate the statistical performance of different combination approaches, extensive Monte Carlo simulations were conducted. Following the simulation design in Biernacka et al. (2011), we selected SNPs in KEGG pathway *maturity_onset_diabetes_of_the_young* which contains 25 genes, to simulate genotypes, based on a total of 5961 observed individuals. The 25 genes covering 599 SNPs were first mapped to 14 chromosomes and then phased using software *fastPHASE* (Scheet and Stephens, 2006). In each of the simulation replicate, 6000 haplotypes were simulated using the *hapsim* library in R (Montana, 2005) based on the haplotype frequencies obtained in the first step. Then pairs of hyplotypes were randomly assigned to 3000 individuals, where the disease status of the *i*th subject *Y_i_* was generated through a Bernoulli distribution, i.e., *Y_i_* ~ *Ber*(*p_i_*) with logit(*p_i_*|***G**_i_*) = α(***G**_i_*), and ***G**_i_* is the genotype vector whose *l*th component ***G**_il_* was coded as 0, 1, or 2 according to the counts of minor alleles at the *l*th SNP (1 ≤ *l* ≤ 599). Based on the 3000 subjects, equal number (*m*) of cases and controls were randomly picked to form the case-control data (*m* = 500, 1000) for each simulation replicate. The type I error (false positive rate) and power were evaluated based on the 0.05 significance level and 1000 simulation replicates.

**Figure 4:**
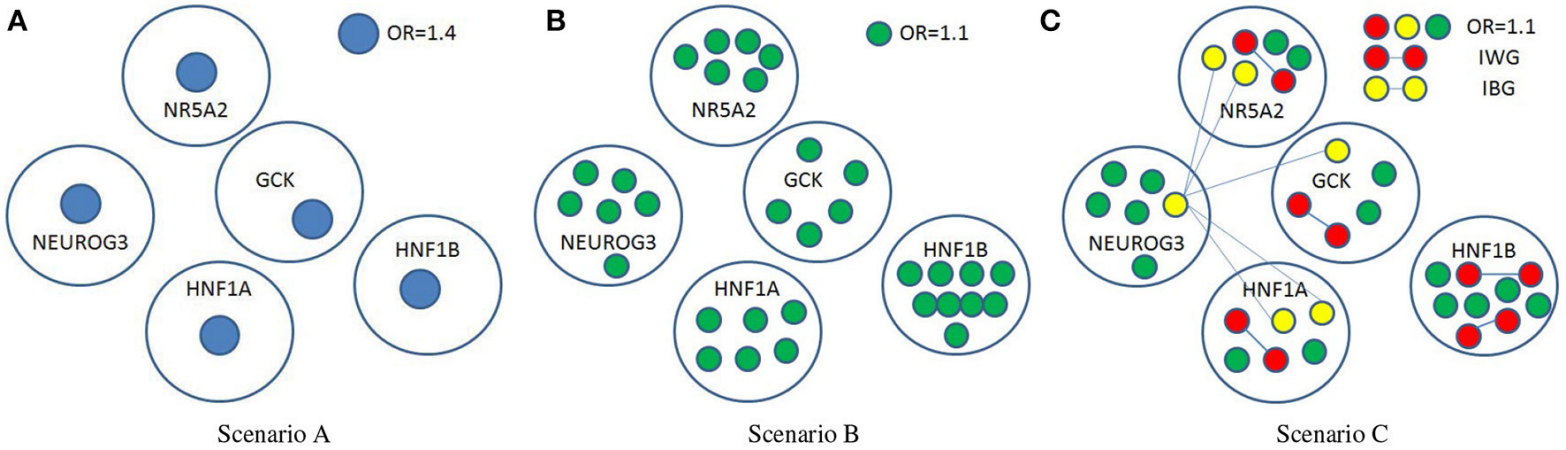
**Three simulation scenarios**. OR, odds ratio; IWG, interactions within a gene; IBG, interactions between genes. **β**_*l*_ = log OR for isolated nodes (SNPs), and Ψ_*st*_ = log OR for any two nodes connected with edge.

Under the null hypothesis of no genetic association between phenotype and pathway, we let α(***G**_i_*) = 0, i.e., logit(*p_i_*) = 0.5, which leads to case:control = 1:1. Under the alternative hypothesis, three scenarios were considered based on the following model,

(3) $$\alpha (G_{i})=G_{i}^{T}\beta +G_{i}^{T}\Psi G_{i},$$

where **β** is a vector with **β**_*l*_ denoting the marginal effect of the *l*th SNP in the pathway, and **Ψ** is a matrix whose (*s*,*t*) entry **Ψ**_*st*_ represents the interaction between the *s*th and *t*th SNP. The three scenarios, which were described in Figure 4 with detailed information listed in Table 2, correspond to different gene actions. Scenario A considers the case in which there is only one moderate marginal effect (odds ratio = 1.4) in each of the five genes. Scenario B assumes there are several small effects (odds ratio = 1.1) in each of the five genes. Scenario C assumes small effects as well as weak interactions within a gene and between genes. In the simulation, only five genes out of 25 were assumed to be associated with the trait. The causal SNPs were fixed in each simulation replicate. The MAF information of the causal SNPs calculated from the real data were given in Supplementary Table 5.

**Table 2:** **List of different simulation scenarios**.

**Gene name**	**Chromosome**	**Gene ID**	**Gene size**	**Scenario A**	**Scenario B**	**Scenario C**
NR5A2	1	2494	95	M	6S	6S (+I)
GCK	7	2645	21	M	5S	5S (+I)
NEUROG3	10	50674	36	M	6S	6S (+I)
HNF1A	12	6927	29	M	6S	6S (+I)
HNF1B	17	6928	44	M	9S	9S (+I)

*S, small effect (OR = 1.1); M, moderate effect (OR = 1.4); +I: with interactions*.

Table 3 summarizes the power and the type I error at the gene level combination using the ARTP and SCC methods. The type I error for MGSEA was rendered to the pathway level analysis. In the table, the results obtained with the SCC method are listed in the parenthesis. The ones with higher power are highlighted with bold font. Clearly the type I error rates for all the five genes under different sample sizes are reasonably controlled by the two methods. For the power, ARTP method achieves higher power than SCC does under scenario A, while SCC method beats ARTP under scenarios B and C for most genes. Note that gene NEUROG3 exhibits different pattern compared to other genes (Table 3) in which ARTP method performs better in Scenario B and C. A summary of the causal SNP's MAF (see Supplementary Table 5) shows that four our of six causal SNPs in this gene have MAF lower than 0.07. Since low MAF generally leads to low test signal, the combined signal under Scenario B/C is mainly determined by the other two SNPs, a case similar to Scenario A where we expect better performance for truncated method (i.e., ARTP).

**Table 3:** Gene-level simulation results with ARTP and SCC under different scenarios^*^.

**case:control**	**Scenario**	**NR5A2**	**GCK**	**NEUROG3**	**HNF1A**	**HNF1B**
500:500	ScenarioA	**0.688** (0.616)	**0.514** (0.461)	**0.632** (0.434)	0.731 (**0.767**)	**0.428** (0.325)
	ScenarioB	0.820 (**0.862**)	0.122 (**0.141**)	**0.159** (0.121)	0.692 (**0.804**)	0.420 (**0.650**)
	ScenarioC	0.505 (**0.581**)	0.116 (**0.169**)	**0.464** (0.354)	0.172 (**0.226**)	0.308 (**0.519**)
	Under the null	0.044 (0.053)	0.045 (0.056)	0.051 (0.053)	0.048 (0.054)	0.051 (0.062)
1000:1000	ScenarioA	**0.966** (0.941)	**0.905** (0.833)	**0.953** (0.80)	**0.976** (0.970)	**0.805** (0.633)
	ScenarioB	0.990 (**0.997**)	0.189 (**0.238**)	**0.341** (0.304)	0.954 (**0.974**)	0.757 (**0.943**)
	ScenarioC	0.836 (**0.880**)	0.205 (**0.288**)	**0.833** (0.688)	0.276 (**0.350**)	0.596 (**0.828**)
	Under the null	0.042 (0.060)	0.044 (0.049)	0.061 (0.048)	0.053 (0.065)	0.046 (0.057)

* *The results using the SCC method are given in the parenthesis while higher power between the two is highlighted with bold font*.

Table 4 shows the power and type I error at the pathway level analysis. The type I error rates are reasonably controlled in all cases. Compared to SCC, ARTP and MGSEA achieve better performance under scenario A. However, under scenario B and C where the joint effect of several SNPs with small marginal effect and/or interactions, SCC is more powerful than ARTP and MGSEA in testing pathway association. Among the three, MGSEA performs the worst. Although SCC is less powerful than the other two under small sample size (2*m* = 1000), it catches up quickly when sample size increases (2*m* = 2000). The simulation results indicate the power gain of the SCC method, in particular under scenarios C when interactions between SNPs are present. To further distinguish the pathway-level power difference under Scenarios A and B with case:control = 1000:1000, as per one reviewer suggestion, we did more simulation by reducing the effect size (i.e., OR). Under Scenario A, ARTP has better power while SCC is quite close to it. Under Scenario B, SCC clearly dominates the other two (see Supplementary Table 6).

**Table 4:** **Pathway-level simulation results under three different scenarios**.

**Method**	**case:control = 500:500**			**case:control = 1000:1000**		
	**ARTP**	**MGSEA**	**SCC**	**ARTP**	**MGSEA**	**SCC**
ScenarioA	**0.852**	0.754	0.757	**1.000**	**1.000**	0.996
ScenarioB	0.755	0.529	**0.784**	0.994	0.971	**0.998**
ScenarioC	0.383	0.294	**0.489**	0.863	0.747	**0.905**
Under the null	0.051	0.042	0.050	0.045	0.043	0.048

*The highest power among the three methods (ARTP, MGSEA and SCC) is highlighted with bold font*.

## 5. Discussion

In this work, we proposed a pathway-based association method by combining single SNP *p*-values in two steps to identify pathways (or gene-sets) associated with a disease trait. Although our model was applied and simulated based on the binary disease trait, it can be utilized for other data types such as quantitative or count trait. The strategy also allows to adjust for the effects of covariates as well as gene-environment interactions. Comparing to other signal combination methods, our methods has the following advantages: (1) Unlike other methods using hard truncation, our method avoids the risk of throwing any important but marginally weak signals while accounting for the correlation structure among the combined signals. For the MGSEA method, a gene signal is represented by the strongest SNP signal within the gene, thus it suffers from power loss by dropping important SNP signals. For GSS, the averaging strategy could lose power when large number of noise SNPs are presented in a set. Although the performance of ARTP might be improved by doing large permutations (e.g., >10,000), it is computational intensive and furthermore, there is no unified criterion on the choice of truncation rank upper bound *T*. The computational load of ARTP would be further enhanced if *T* is large (e.g., causal SNPs/genes are dense in gene/pathway); (2) The scaled chi-square approximation can achieve small *p*-values which is computationally burdensome for other permutation based methods such as ARTP. Hence, SCC is computationally efficient and small *p*-values can be achieved without large scale permutations; (3) As revealed by simulation studies (Table 4), SCC exhibits great power than other two methods when there exist interactions between SNPs in a gene set.

We applied our method to two cohort studies of type 2 diabetes with pathways defined in KEGG databases. Several sex-specific patterns were observed. Firstly, both the gene- and pathway-level scan results showed that there was not much signal overlap between the female and male population, as also revealed by applying other tools (e.g., ARTP, MGSEA, and GSS). Secondly, more pathways were enriched in female population comparing to male population. SCC exhibited greater performance in detecting associated pathways, especially for the curated KEGG T2D pathway *type II diabetes mellitus* which cannot be detected by other methods such as ARTP, MGSEA, and GSS. In addition, gene TCF7L2 in female population also shows significance after *p*-value combination, while no single SNP in this gene shows significance. This clearly demonstrates the power of signal combination to identify SNPs that function jointly but could be missed by marginal analysis. We also did the QQ plot of the *p*-values (results not shown). The gene-based *p*-value QQ plots showed a reasonable pattern at different significance levels. But for the pathway-based *p*-value QQ plots, there is a moderate departure from the expected. This is due to the fact of dependent *p*-values since many pathways share common genes and the pathway-based *p*-values are not completely independent.

Our simulation studies showed reasonable control of false positive rate of the method, on either gene- or pathway-level combination. Simulation studies also revealed that the optimal choice of combination methods depends on the underlying true disease model. As what we expected, under scenario A where only one SNP with moderate effect functions in each of the five genes, ARTP and MGSEA were more powerful. This is because the default *T* is 1 for SNP-level and 10 for gene-level in ARTP, and MGSEA only picks the most significant signal at each level. Under scenario B and C where there exist several SNPs with small marginal effects and interactions contributing to a disease risk, SCC obtained better performance, benefitted from no truncation (i.e., no information dropping). In reality, the true model is generally unknown although scenarios B and C are more likely. We suggest users to apply different methods and pool top signals for further functional validation.

### Conflict of interest statement

The authors declare that the research was conducted in the absence of any commercial or financial relationships that could be construed as a potential conflict of interest.
