# Supplementary material for: A set-based association test identifies sex-specific gene sets associated with type 2 diabetes
Source: Front Genet. 2014 Nov 12;5:395. doi: 10.3389/fgene.2014.00395 (PMC4228910; doi:10.3389/fgene.2014.00395)
Supplement: Supplementary file 1 [file Table1.PDF]

Frontiers in Genetics

Research Article
16 October 2014

Supplementary Material: A Set-based Association Test Identifies Sex-specific Gene Sets Associated with Type 2 Diabetes

Tao He [1], Ping-Shou Zhong [1] and Yuehua Cui [1,2*]

[1] *Department of Statistics and Probability, Michigan State University, East Lansing, Michigan, USA*
[2] *Division of Medical Statistics, School of Public Health, Shanxi Medical University, Taiyuan, Shanxi, China*

Correspondence*:
Yuehua Cui
619 Red Cedar Rd. Rm C432, East Lansing, MI, 48824, USA , cui@stt.msu.edu

Supplementary Table 1. List of top signals at the gene level analysis using methods SCC, ARTP and GSS.

Method	Top gene level signals*
	female group (top 5 signals)
SCC	**BST1**(4, 1.0e-7), **PIGQ**(16, 2.2E-6), IL18RAP(2, 9.1e-6), **TCF7L2**(10, 1.1e-5), ALG1(16, 4.6e-5)
ARTP	KCNQ1(11, 1.0e-3), **TCF7L2**(10, 1.0e-3), FGF23(12, 1.0e-3), IL18R1(2, 1.0e-3), **PIGQ**(16, 1.0e-3)
GSS	FAM200B(4, 0.024), FBXL5(4, 0.026), **BST1**(4, 0.055), **TCF7L2**(10, 0.796), MIEF2(17, 0.876)
	male group (top 6 signals)
SCC	**TCF7L2**(10,7.1e-12), **SNRNP200**(2,2.9e-7), **MCM3**(6,7.0e-7), GYS2(12,5.9.0e-6), **DUSP2**(2,8e-6), ABO(9,1.1e-5)
ARTP	CRK(17,1.0e-7), ADRA2B(2,1.0e-7), **DUSP2**(2,1.0e-7), **SNRNP200**(2,1.0e-7), **MCM3**(6,1.0e-7), **TCF7L2** (10,1.0e-7)
GSS	**TCF7L2**(10,0),STARD7(2,0.053),CIAO1(2,0.074),TMEM127(2,0.074),**SNRNP200**(2,0.124),STARD7-AS1(2,0.139)

* signal format: GeneName (chromosome, p-value). Common signals shared by at least two methods are highlighted in bold font.

Supplementary Table 2. Top signals by pathway-level analysis using the ARTP method.

Pathway	No. of genes	p-value
Top signals in female group		
HOMOLOGOUS RECOMBINATION	27	5.00e-3
**BASAL CELL CARCINOMA**	55	6.00e-3
**INSULIN SIGNALING PATHWAY**	130	1.20e-2
GLYCOSAMINOGLYCAN DEGRADATION	20	1.50e-2
**COLORECTAL CANCER**	61	1.70e-2
**WNT SIGNALING PATHWAY**	147	2.10e-2
MATURITY ONSET DIABETES OF THE YOUNG	25	2.10e-2
VIBRIO CHOLERAE INFECTION	52	2.40e-2
NICOTINATE AND NICOTINAMIDE METABOLISM	24	2.40e-2
TGF BETA SIGNALING PATHWAY	86	2.50e-2
GLYCOSPHINGOLIPID BIOSYNTHESIS GANGLIO SERIES	26	2.80e-2
LYSOSOME	114	2.80e-2
AXON GUIDANCE	124	3.00e-2
ADIPOCYTOKINE SIGNALING PATHWAY	64	3.10e-2
GLYCOLYSIS GLUCONEOGENESIS	59	3.30e-2
**CELL CYCLE**	122	3.30e-2
ACUTE MYELOID LEUKEMIA	53	3.60e-2
Top signals in male group		
**COLORECTAL CANCER**	61	9.99e-4
**CELL CYCLE**	122	9.99e-4
MAPK SIGNALING PATHWAY	256	2.00e-3
**WNT SIGNALING PATHWAY**	147	3.00e-3
ADHERENS JUNCTION	73	3.00e-3
**BASAL CELL CARCINOMA**	55	3.00e-3
PANCREATIC CANCER	66	5.00e-3
**INSULIN SIGNALING PATHWAY**	130	6.00e-3
MELANOGENESIS	100	7.00e-3
CYSTEINE AND METHIONINE METABOLISM	33	7.00e-3
GLYCEROLIPID METABOLISM	46	9.00e-3

Common pathways shared in the two groups are highlighted in bold font.

Supplementary Table 3. Top signals by pathway-level analysis using the MGSEA method.

Pathway	No. of genes	p-value
Top signals in female group		
**WNT SIGNALING PATHWAY**	147	0.001
CITRATE CYCLE TCA CYCLE	30	0.005
LYCOSYLPHOSPHATIDYLINOSITOL GPI ANCHOR BIOSYNTHESIS	75	0.008
ADHERENS JUNCTION	73	0.009
GRAFT VERSUS HOST DISEASE	36	0.015
GLYCOSPHINGOLIPID BIOSYNTHESIS LACTO AND NEOLACTO SERIES	15	0.016
GLYCOSAMINOGLYCAN DEGRADATION	20	0.016
T CELL RECEPTOR SIGNALING PATHWAY	104	0.021
BASE EXCISION REPAIR	33	0.025
PARKINSONS DISEASE	111	0.029
ALZHEIMERS DISEASE	156	0.031
NATURAL KILLER CELL MEDIATED CYTOTOXICITY	129	0.042
Top signals in male group		
COLORECTAL_CANCER	61	0.008
JAK_STAT_SIGNALING_PATHWAY	147	0.016
HEMATOPOIETIC_CELL_LINEAGE	80	0.028
VALINE_LEUCINE_AND_ISOLEUCINE_DEGRADATION	43	0.033
FATTY_ACID_METABOLISM	41	0.033
AUTOIMMUNE_THYROID_DISEASE	48	0.035
**WNT_SIGNALING_PATHWAY**	147	0.037
REGULATION_OF_ACTIN_CYTOSKELETON	202	0.038

Supplementary Table 4. Top signals by pathway-level analysis using the GSS method.

Pathway	No. of genes	p-value
Top signals in female group		
TYPE I DIABETES MELLITUS	41	0.083
BASAL CELL CARCINOMA	55	0.376
COLORECTAL CANCER	61	0.578
Top signals in male group		
FATTY ACID METABOLISM	41	0.704
COLORECTAL CANCER	61	0.925

Supplementary Table 5. MAF information of causal SNPs in simulation.

Gene name	Scenario A	Scenario B/C
NR5A2	0.34	0.35, 0.37, 0.37, 0.23, 0.34
GCK	0.18	0.14, 0.48, 0.17, **0.07**, 0.18
NEUROG3	0.27	**0.06**, 0.27, **0.07**, **0.07**, **0.06**, 0.35
HNF1A	0.35	0.35, 0.11, 0.40, 0.37, 0.32, 0.33
HNF1B	0.23	0.45, 0.19, 0.45, 0.16, 0.23, 0.19, 0.24, 0.18, 0.49

MAF$\leq$ 0.07 are highlighted in bold font.

Table 6. Pathway-level simulation results under Scenarios A and B with case:control=1000:1000*

Scenario	ARTP	MGSEA	SCC
A+	**0.527**	0.390	0.523
B+	0.346	0.223	**0.445**

* Power comparisons were made among the three methods under a certain scenario, where the highest one is highlighted in bold font.
+ Scenarios A and B are designed with reduced ORs (1.2 in A and 1.05 in B), in parallel to the original Scenarios A and B
